# Severe hypoglycaemia is associated with increased risk of adverse cardiovascular complications in adults with type 1 diabetes: risk mitigation using intermittently scanned continuous glucose monitoring

**DOI:** 10.1007/s00125-025-06438-y

**Published:** 2025-04-24

**Authors:** Katarina Eeg-Olofsson, David Nathanson, Tim Spelman, Mattias Kyhlstedt, Alexander Seibold, Fleur Levrat-Guillen, Jan Bolinder

**Affiliations:** 1 Sahlgrenska University Hospital and Department of Molecular & Clinical Medicine, University of Gothenburg, Gothenburg, Sweden; 2 Department of Medicine, Karolinska University Hospital Huddinge, Karolinska Institute, Stockholm, Sweden; 3 Synergus RWE AB, Åkersberga, Sweden; 4 Abbott Diabetes Care, Wiesbaden, Germany; 5 Abbott Laboratories Ltd, Maidenhead, UK

**Keywords:** Cardiovascular, Hospitalisation, Intermittently scanned CGM, Major adverse cardiac events, Type 1 diabetes

## Abstract

**Aims/hypothesis:**

It has been proposed that severe hypoglycaemia events (SHE) may increase the risk of adverse CVD complications in adults with type 1 diabetes. The aim of this study was to evaluate the risk of CVD complications following SHE in a large cohort of adults with type 1 diabetes, and to compare the risk of post-SHE CVD complications for users of intermittently scanned continuous glucose monitoring (isCGM) vs users of blood glucose monitoring (BGM).

**Methods:**

This comparative retrospective cohort study used data from the Swedish National Diabetes Register and the Swedish National Patient Register. We identified people with type 1 diabetes who had a hospitalisation for CVD complications. Rates of hospitalisation were compared between those with an index SHE and those without, and within isCGM or BGM subgroups. The study baseline was date of the first SHE prior to the isCGM index date.

**Results:**

We identified 14,829 adults with type 1 diabetes with up to 2 years of follow-up, of which 1313 had an index SHE. In the full cohort, the relative rate of hospitalisations for CVD complications was 2.06-fold (95% CI 1.48, 2.85) in those with prior SHE. Of these 1313 participants with prior SHE, 970 were using isCGM and 343 were using BGM. Hospitalisations for post-SHE CVD complications were significantly lower for isCGM users (5.40 per 100 person-years of follow-up; 95% CI 4.59, 6.31) compared with BGM control participants (14.23 per 100 person-years of follow-up; 95% CI 11.95, 16.82), which represents a 78% relative reduction in rates of post-SHE CVD complications for isCGM users (relative rate 0.22; 95% CI 0.11, 0.43; *p*<0.001), after adjustment for confounders.

**Conclusions/interpretation:**

In adults with type 1 diabetes, SHE is associated with an increased risk of hospitalisation for adverse CVD complications. This risk is significantly reduced in isCGM users compared with BGM control participants.

**Graphical Abstract:**

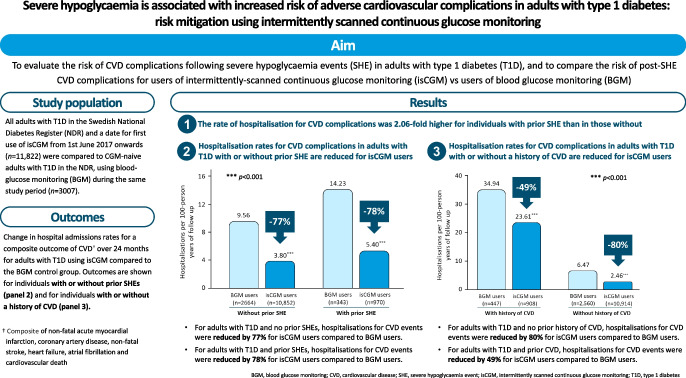

**Supplementary Information:**

The online version contains peer-reviewed but unedited supplementary material available at 10.1007/s00125-025-06438-y.


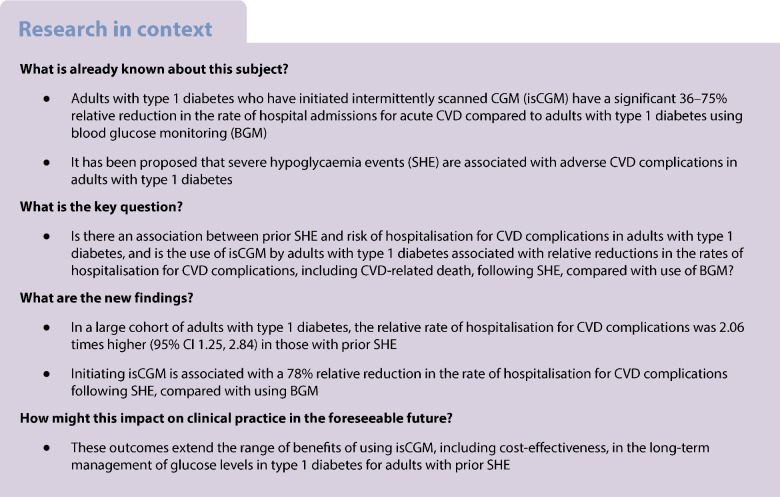


## Introduction

Despite advances in glycaemic management, people with type 1 diabetes remain at higher risk of CVD and CVD-related mortality than people without diabetes [1]. Data from the Swedish National Diabetes Register (NDR) reveal that people with type 1 diabetes and HbA_1c_ ≤52 mmol/mol (≤6.9%) have a twofold greater risk of CVD death than a matched population without diabetes [2]. For individuals with type 1 diabetes and HbA_1c_ ≥82 mmol/mol (≥9.7%), this risk is estimated to be 8–10-fold [2].

The Diabetes Control and Complications Trial (DCCT) and the Epidemiology of Diabetes Interventions and Complications (EDIC) study have shown that more-intensive glucose control for people with type 1 diabetes is associated with a 42% reduced risk of any cardiovascular event and a 57% risk reduction of death from CVD [3] over a mean of 6.5 years. However, prior severe hypoglycaemia events (SHEs) are reported to increase the risk of adverse CVD complications in people with type 1 diabetes [4], and SHEs are independently associated with accelerated atherosclerosis, increased risk of CVD events and all-cause mortality [5–7]. Acute or recurrent hypoglycaemia episodes are known to induce proinflammatory mediators, platelet activation and markers of vascular endothelial stress in type 1 diabetes [8, 9].

Continuous glucose monitoring (CGM) technologies have enabled people with type 1 diabetes to significantly improve their glucose levels and reduce exposure to hypoglycaemia, compared with using blood glucose monitoring (BGM) test strips [10–13]. We have previously shown that the use of intermittently scanned CGM (isCGM) is associated with lowered HbA_1c_ within 6 months of initiation, and that these levels persist for at least 2 years of follow-up, in addition to lower rates of SHEs compared with BGM control participants [14]. Using data from the NDR, linked with hospital admissions data from the Swedish National Patient Register (NPR), we have also reported that, compared with a matched cohort of BGM users, isCGM users with type 1 diabetes have a 36–75% relative reduction in rates of hospitalisation for CVD events, including acute myocardial infarction (AMI), stroke, heart failure and atrial fibrillation, as well as a relative reduction in hospitalisation for acute diabetes events (68% relative reduction in rates of SHE and 45% relative reduction for diabetic ketoacidosis) over a 24-month follow-up period, together with an HbA_1c_ reduction of approximately 0.3% [15].

Mechanistically, it seems unlikely that reductions in CVD events were linked to improved glucose control, considering the short observation period and modest difference in HbA_1c_. Instead, we speculate that the reductions in CVD events may be associated with the observed risk reduction for SHEs. Studies in healthy individuals and in people with type 1 diabetes indicate that acute hypoglycaemia leads to increased proinflammatory cytokines, mobilisation of inflammatory leukocytes and increased platelet reactivity [16–18]. Such consequences are hypothesised to promote atherogenesis and plaque instability in people with diabetes. To investigate this hypothesis, the current study uses linked data from the NDR and NPR to examine the association between prior SHE and hospital admissions for non-fatal CVD complications and CVD-related death in adults with type 1 diabetes, and compared the rate of CVD complications following a prior SHE for isCGM users vs a control group of BGM users.

## Methods

The Swedish NDR has >90% coverage of adults with type 1 diabetes in Sweden [19]. As diabetes type is stored at the person level in the NDR and is updated in the last recorded observation, there is a low risk for errors in this variable. All adults (≥18 years old) with a clinical diagnosis of type 1 diabetes and with a diabetes clinic visit recorded in the NDR after 1 January 2014 and up to 31 July 2023, and first recorded use (index date) of isCGM from 1 June 2017 onwards, were included. A control group of adults with type 1 diabetes was identified, comprising all individuals who were CGM-naive during the study inclusion period (see below). Adults for whom any prior use of CGM devices was recorded in the NDR were excluded. For included individuals, data were collected over a 24-month follow-up period.

### Data completeness

New incident users of isCGM among adults with type 1 diabetes registered in the NDR were assessed for prior use of CGM and a SHE, defined as an event requiring third-party assistance or hospital admission, recorded in the NDR prior to the isCGM index date, as well as history of CVD, defined as any CVD complication recorded in the NPR prior to the baseline SHE event. For this study, we applied a six-point definition of CVD complications as a composite of non-fatal AMI, coronary artery disease, non-fatal stroke, heart failure or atrial fibrillation, as the primary cause of hospitalisation in each case, or cardiovascular death. Missing values in this category occur if the information is unknown, or if the assessment was not conducted or recorded by the responsible healthcare professional.

### Incident use of isCGM in individuals with type 1 diabetes

**Fig. 1:**
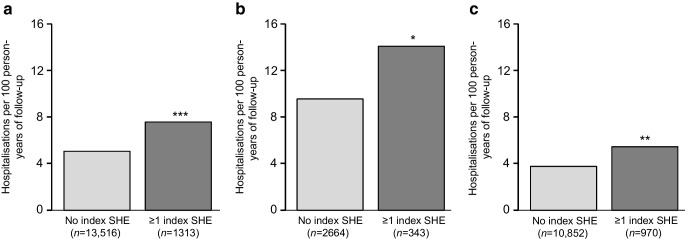
Rate of hospitalisation for CVD complications in adults with T1D with or without prior experience of SHE. Negative binomial regression was used to compare the relative rates of CVD complications for people with T1D with and without prior experience of SHE. (**a**) All adults with T1D; (**b**) adults with T1D using BGM; (**c**) adults with T1D using isCGM. PS-IPTW was used to adjust for differences in confounders at baseline between the groups. Relative rates of hospitalisations are shown. Asterisks indicate statistically significant differences compared to the group with no SHE: *p<0.05, **p<0.01, ***p<0.001. T1D, type 1 diabetes

All adults with type 1 diabetes and an NDR index date for first use of isCGM from 1 June 2017 to 31 July 2023 were identified within each calendar year, and assessed for prior use of any CGM device and a SHE recorded in the NDR prior to the isCGM index date, as well as a history of CVD. The baseline was defined as the date of the first recorded SHE in the NDR prior to the 1 June 2017 isCGM index date, which was also used as the baseline date for non-SHE individuals (see electronic supplementary material [ESM] Fig. 1).

### BGM control group

All CGM-naive adults with type 1 diabetes recorded at the person level in the NDR during the same study period (ESM Fig. 1), with HbA_1c_ values recorded within the same 3–8-month baseline window and follow-up period as the isCGM group and assessed for SHE and history of prior CVD, were used as a BGM-only control group.

### Hospital admission rates for CVD complications in incident isCGM users and BGM control participants

Hospital admission data for new incident isCGM users and BGM control participants with type 1 diabetes were extracted from the NPR [20], which provides administrative data on hospital admissions and inpatient care, with associated diagnoses and procedures coded according to the Swedish version of ICD- 10 (https://icd.who.int/browse10/2019/en) (ESM Table 1). The CVD complications reported here were the primary diagnosis for hospital admission (ESM Table 1). Within the isCGM and BGM cohorts, the relative reduction in rates of hospitalisation for CVD complications between those with an index SHE and those without was assessed.

### Statistical analysis

Categorical variables were summarised using frequency and percentage. Continuous variables were summarised using mean and SD or median and IQR, as appropriate. Propensity score-based inverse probability of treatment weighting (PS-IPTW) negative binomial regression was used to compare rates of hospital admission for CVD complications between isCGM users and BGM control participants, offset by follow-up duration. The propensity score weighting was derived using a logistic regression in which case vs control status was set as the dependent outcome variable, and age, sex, BMI, baseline HbA_1c_, use of a continuous subcutaneous insulin infusion (CSII) pump, lipid profile, renal function, smoking status, physical activity, pre-baseline comorbidity and diabetic complications were defined as the independent explanatory variables (Table 1 and ESM Tables 2–4). The performance of the weighting (i.e. how balanced the two groups were) was assessed through derivation and analysis of weighted standardised differences. Hospital admission counts were assessed for overdispersion. For all analyses, a *p* value <0.05 was considered significant. All analyses were performed using Stata version 17 (StataCorp) and R version 4.2.3 (R Foundation for Statistical Computing).

**Table 1:** Baseline characteristics for all adults with type 1 diabetes, with and without a prior SHE

Characteristic at baseline registration in dataset	Adults with T1D and prior SHE (*n*=1313)	Adults with T1D without prior SHE (*n*=13,516)	Standardised difference	Weighted baseline characteristics		Weighted standardised difference
				Adults with T1D and prior SHE (*n*=1313)	Adults with T1D without prior SHE (*n*=13,516)	
Age (years)	54.29 ± 18.66	52.79 ± 18.52	0.081	53.96 ± 18.54	53.02 ± 18.22	0.051
Sex						
Female	554 (42.2)	5620 (41.6)	0.012	552 (42.0)	5626 (41.6)	0.007
Male	759 (57.8)	7896 (58.4)		761 (58.0)	7890 (58.4)	
BMI (kg/m^2^)	26.40 ± 3.44	26.35 ± 3.80	0.013	26.39 ± 3.42	26.36 ± 3.58	0.009
HbA_1c_ (mmol/l)	63.2 ± 13.7	62.5 ± 13.9	0.051	63.0 ± 13.5	62.6 ± 13.6	0.024
HbA_1c_ (%)	7.93 ± 1.26	7.87 ± 1.27		7.91 ± 1.24	7.88 ± 1.25	
Diabetes duration (years)	25.78 ± 17.22	22.24 ± 16.68	0.209	24.23 ± 16.77	22.49 ± 16.98	0.103
Insulin pump users	130 (9.9)	1238 (9.2)	0.025	125 (9.5)	1246 (9.2)	0.019
SBP (mmHg)	129.78 ± 13.70	129.10 ± 13.19	0.050	129.55 ± 13.65	129.31 ± 13.21	0.018
DBP (mmHg)	73.96 ± 8.06	74.44 ± 7.95	−0.060	74.10 ± 7.94	74.41 ± 7.95	−0.039
LDL-cholesterol (mmol/l)	2.45 ± 0.64	2.48 ± 0.68	−0.049	2.45 ± 0.63	2.45 ± 0.64	0.000
HDL-cholesterol (mmol/l)	1.58 ± 0.38	1.58 ± 0.41	0.006	1.58 ± 0.38	1.58 ± 0.38	0.000
Triglycerides (mmol/l)	1.21 ± 0.56	1.20 ± 0.68	0.011	1.21 ± 0.57	1.21 ± 0.57	0.000
Total cholesterol (mmol/l)	4.46 ± 0.73	4.48 ± 0.79	−0.027	4.46 ± 0.75	4.48 ± 0.76	−0.026
Creatinine (µmol/l)	83.10 ± 36.69	79.65 ± 37.55	0.093	82.57 ± 36.55	80.94 ± 36.91	0.044
eGFR (ml/min per 1.73 m^2^)	86.79 ± 24.15	89.83 ± 23.20	−0.128	87.46 ± 23.95	88.73 ± 23.52	−0.054
Albuminuria						
No	1157 (88.1)	12,164 (90.0)	0.060	1160 (88.3)	12,161 (90.0)	0.055
Previous	27 (2.1)	199 (1.5)		25 (1.9)	203 (1.5)	
Microalbuminuria	92 (7.0)	889 (6.6)		90 (6.9)	890 (6.6)	
Macroalbuminuria	37 (2.8)	264 (2.0)		38 (2.9)	262 (1.9)	
Physical activity						
Never	146 (11.1)	1257 (9.3)	−0.074	141 (10.7)	1262 (9.3)	−0.066
Less than once a week	173 (13.2)	1646 (12.2)		171 (13.0)	1650 (12.2)	
Regularly (1–2 times a week)	280 (21.3)	2648 (19.6)		288 (21.9)	2655 (19.6)	
Regularly (3–5 times a week)	350 (26.7)	4131 (30.6)		357 (27.2)	4128 (30.5)	
Daily	364 (27.7)	3834 (28.4)		356 (27.1)	3821 (28.3)	
Ischaemic heart disease	137 (10.4)	1218 (9.0)	−0.048	135 (10.3)	1224 (9.1)	−0.041
Retinopathy	911 (69.4)	8435 (62.4)	−0.148	904 (68.8)	8467 (62.6)	−0.125
Stroke	91 (6.9)	618 (4.6)	−0.101	86 (6.5)	623 (4.6)	−0.091
Smoker	162 (12.3)	1548 (11.5)	−0.027	160 (12.2)	1555 (11.5)	−0.024

Values for categorical variables are *n* (%); values for continuous variables are means ± SD

DBP, diastolic BP; SBP, systolic BP; T1D, type 1 diabetes

### Ethics approval

The study protocol was approved by the Swedish Ethical Review Authority (Dnr 2021–02886).

## Results

We identified 14,829 adults with type 1 diabetes who fulfilled the inclusion criteria, of whom 11,822 had initiated isCGM within the study period, along with 3007 adults with type 1 diabetes who were using BGM (control participants). Across the total cohort, 1313 (8.9%) had reported at least one index SHE during the study period; 13,516 (91.1%) did not report a prior SHE. The baseline characteristics for each group before and after PS-IPTW are shown in Table 1. After weighting, these two cohorts of adults with type 1 diabetes were well balanced, with weighted standardised differences of less than 0.10 for 18 of 20 covariates, indicating a negligible difference in the mean or prevalence of these covariates between the groups, which are therefore considered to have no confounding influence [21]. The weighted standardised differences for diabetes duration (0.103) and retinopathy (−0.125) were below 0.20 (Table 1), which indicates only a small residual confounding [22] due to these covariates after weighting; this is considered unlikely to influence the outcomes. Of the adults with type 1 diabetes who had experienced a prior SHE, 970 were isCGM users and 343 were BGM users. After PS-IPTW, the baseline characteristics of these two study groups were also well balanced (ESM Table 2), with weighted standardised differences less than 0.10 for seven of the 20 covariates, indicating no confounding influence [21] for these covariates. Of the remaining 13 covariates, 12, including age, use of insulin pump therapy and history of ischaemic heart disease, showed only a small residual confounding [22], with weighted standardised differences below 0.20. Only for one covariate (serum creatinine) was the weighted standard difference slightly higher than 0.20 (ESM Table 2). A double robust method, using additional variable adjustment in the regression analysis, was used to minimise the effect of remaining imbalance after weighting [23]. The baseline characteristics for isCGM users with and without prior SHE are shown in ESM Table 3, and those for BGM control participants are shown in ESM Table 4. PS-IPTW indicated no confounding influence for the majority of baseline covariates between the groups with or without prior SHEs, or small residual confounding for the remainder that is unlikely to influence outcomes.

### Hospital admissions for CVD complications among adults with type 1 diabetes with or without prior experience of SHE

For adults with type 1 diabetes, the rate of hospital admission for CVD complications was calculated using data from the NPR for individuals with or without a prior SHE (Table 2 and Fig. 1a). Those with no prior SHE had a rate of 5.04 (95% CI 4.85, 5.23) hospitalisations per 100 person-years of follow-up compared with 7.58 (95% CI 6.74, 8.49) for the group with at least one SHE (relative rate 2.06; 95% CI 1.48, 2.85; *p*<0.001).

**Table 2:** Hospital admissions for CVD complications among adults with type 1 diabetes, with and without a prior SHE episode, by glucose-monitoring method

	Adults with T1D	CVD hospital admissions	Person-years of follow-up	CVD hospitalisation event rate^a^ (95% CI)	Incident rate *p* value	Relative rate^b^ (95% CI)	*p* value^c^
All users							
Without prior SHE	13,516	2697	53,533.90	5.04 (4.85, 5.23)	<0.001	2.06 (1.48, 2.85)	<0.001
With prior SHE	1313	296	3907.51	7.58 (6.74, 8.49)			
BGM users							
Without prior SHE	2664	1099	11,502.31	9.56 (9.00, 10.14)	<0.001	1.80 (1.08, 2.99)	0.023
With prior SHE	343	137	962.65	14.23 (11.95, 16.82)			
isCGM users							
Without prior SHE	10,852	1598	42,031.59	3.80 (3.62, 3.99)	<0.001	1.89 (1.25, 2.84)	0.002
With prior SHE	970	159	2944.86	5.40 (4.59, 6.31)			

^a^Hospital event rate per 100 person-years of follow-up

^b^Relative rates for admission to hospital for adults with T1D with a prior SHE, compared to those without a prior SHE. PS-IPTW negative binomial regression was used to calculate rates of hospital admission for diabetes-related events per 100 person-years of cumulative follow-up

^c^Probability for change in relative rates for hospital admission for adults with T1D with a prior SHE compared to without a prior SHE

T1D, type 1 diabetes

For adults with type 1 diabetes using BGM and no prior SHE, the hospital admission rate was 9.56 (95% CI 9.00, 10.14) per 100 person-years of follow-up (Table 2 and Fig. 1b), compared with 14.23 (95% CI 11.95, 16.82) for BGM users with at least one prior SHE (relative rate 1.80; 95% CI 1.08, 2.99; *p*=0.023). Among adults with type 1 diabetes using isCGM, those with no previous SHE had a hospital admission rate for CVD complications of 3.80 (95% CI 3.62, 3.99) per 100 person-years of follow-up (Table 2 and Fig. 1c), compared with 5.40 (95% CI 4.59, 6.31) for the group with at least one SHE (relative rate 1.89; 95% CI 1.25, 2.84; *p*=0.002).

**Fig. 2:**
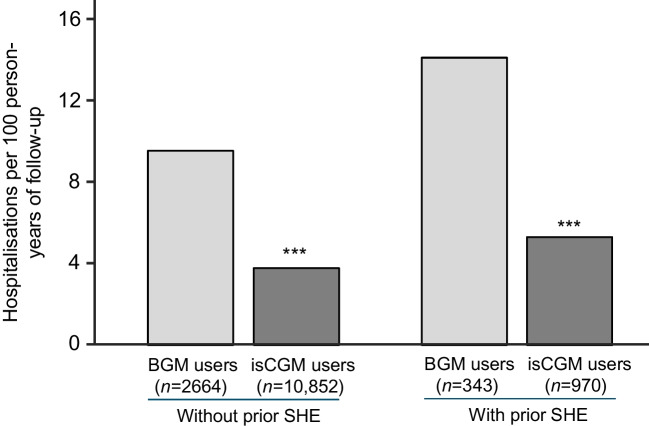
Hospitalisation rate for CVD complications in adults with T1D with or without prior SHE, by glucose-monitoring method. Negative binomial regression was used to compare the relative rates of CVD complications following SHE in isCGM users relative to BGM users. PS-IPTW was used to adjust for differences in confounders at baseline between the groups. Relative rates of hospitalisations are shown. Asterisks indicate statistically significant differences compared with the BGM group: ***p<0.001. T1D, type 1 diabetes

A comparison of hospital admission rates for CVD complications among adults with type 1 diabetes and previous SHE who used isCGM vs BGM is shown in Fig. 2. The risk of hospitalisation for CVD complications for isCGM users and experience of SHE was significantly lower than that for BGM users (5.40 vs 14.23 per 100 person-years of follow-up), with a 78% relative reduction in the rate of admission for CVD complications (relative rate 0.22; 95% CI 0.11, 0.43; *p*<0.001). In the subgroup without prior SHE, isCGM use was associated with a 77% relative reduction in rates of CVD complications compared with BGM users (relative rate 0.23; 95% CI 0.18, 0.28; *p*<0.001). When we used a composite outcome that is more typical of major adverse cardiac events, consisting of a composite of AMI, stroke or cardiovascular death, we saw the same pattern (ESM Fig. 2), with a 63% relative reduction in rates of CVD complications for isCGM users with a prior SHE compared to BGM users with a prior SHE (relative rate 0.37; 95% CI 0.18, 0.75; *p*=0.006). Using this narrower definition of cardiovascular complications for adults without prior SHE (ESM Fig. 2), isCGM users had a 70% relative reduction in the rates of CVD complications compared with BGM users (relative rate 0.30; 95% CI 0.26, 0.38; *p*<0.001).

### Hospital admissions for CVD complications among adults with type 1 diabetes with or without a history of CVD

**Fig. 3:**
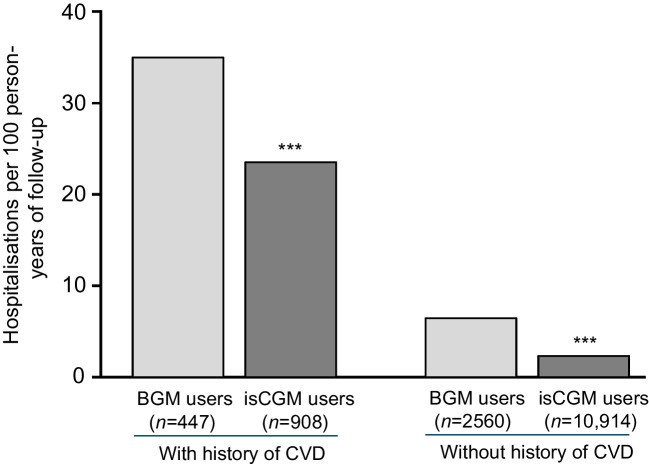
Hospitalisation rate for CVD complications in adults with T1D with or without history of CVD, by glucose-monitoring method. Negative binomial regression was used to compare the relative rates of CVD complications in isCGM users relative to BGM users, among adults with T1D with or without a history of CVD. PS-IPTW was used to adjust for differences in confounders at baseline between the groups. Relative rates of hospitalisations are shown. Asterisks indicate statistically significant differences compared with the BGM group: ***p<0.001. T1D, type 1 diabetes

Among the full cohort of adults with a prior SHE, we identified those with a history of CVD and those without, and compared the rate of hospitalisation for CVD complications for isCGM users vs BGM users (Fig. 3). For adults with type 1 diabetes using BGM who had a history of CVD, the rate of hospitalisation for CVD complications was 34.94 (95% CI 32.02, 38.05) per 100 person-years of follow-up compared with 23.61 (95% CI 21.93, 25.39) for isCGM users who had a history of CVD (*p*<0.001). This equates to a 49% relative reduction in rates of admission for CVD complications for isCGM users. Adults with type 1 diabetes but no history of CVD experienced much lower rates of hospital admissions for CVD complications (Fig. 3). For BGM users, the rate was 6.47 (95% CI 6.00, 6.97) per 100 person-years of follow-up, compared with 2.46 (95% CI 2.31, 2.61) for isCGM users (*p*<0.001), which is an 80% relative reduction in rates.

## Discussion

This comparative retrospective study demonstrates that prior SHE is associated with an increased risk of hospitalisation for CVD complications in adults with type 1 diabetes, and reveals important changes in the risk of post-SHE CVD complications for isCGM users. Our data confirm that risk of hospital admission for CVD complications is approximately doubled among adults with type 1 diabetes who had a previous SHE, compared to those without a recorded SHE (Fig. 1a). The highest rate of hospital admissions for CVD complications was in the cohort of adults with type 1 diabetes who were BGM users (Fig. 1b). Adults with type 1 diabetes who were isCGM users had a lower rate of hospitalisation for CVD complications (Fig. 1c), in both the group with and those without a prior SHE. In keeping with the wider observation that a prior SHE episode confers an approximately doubled risk of hospitalisation for CVD complications, the post-SHE cohort of isCGM users exhibited a 1.89-fold relative increase in rate of admission for CVD complications (Fig. 1c).

The most notable outcome is that isCGM users with a prior SHE had a greatly reduced frequency of admission for CVD complications compared to BGM users with a previous SHE (Fig. 2). Equally notable are the significant reductions in risk of CVD complications for isCGM users with no prior SHE, compared with BGM users. Use of isCGM has been shown to reduce less-severe hypoglycaemia in type 1 diabetes [12, 13], which may contribute to the lower incidence of CVD complications shown here. These outcomes were replicated when using a narrower composite definition of CVD complications, including AMI, stroke and cardiovascular death (ESM Fig. 2). This aligns with our previous study showing that isCGM users have a significant relative reduction in rates of hospital admissions for cardiovascular complications of type 1 diabetes, compared with BGM control participants, including stroke, AMI, heart failure and atrial fibrillation [15]. Our subgroup analysis showed that risk of CVD complications was significantly higher in the subgroup of adults with type 1 diabetes and a history of CVD (Fig. 3), and that using isCGM significantly reduced this risk.

Our study has several noteworthy outcomes. These include demonstration of the association of at least one prior SHE with a significantly increased risk of CVD complications for people with type 1 diabetes, using the composite endpoint of hospitalisation for CVD complications, rather than CVD mortality alone. A 2014 study using NDR data to show an association of prior SHE with CVD mortality in type 1 diabetes [4] concluded that, although prior SHE was associated with reduced survival following a major CVD event, it was not associated with an increased risk of a subsequent fatal CVD event. Our data, using hospital admissions data, suggest an association between prior SHE and an increased risk of composite CVD complications, for people with type 1 diabetes using either BGM or isCGM. This finding must be reconciled with the EURODIAB study outcomes, which did not find that prior SHE increased the risk of fatal or non-fatal cardiovascular events [24] and the finding of a prospective observational study on two cohorts of people with type 1 diabetes, one Danish (*n*=269) and one Dutch (*n*=482), neither of which found an association between episodes of SHE or impaired awareness of hypoglycaemia and CVD mortality when not part of a composite outcome [25]. The difference in outcomes between the previous NDR study on CVD mortality following SHE [4] and the current study may also reflect different selection criteria and reporting aims. The 2014 study included risk factor data from the NDR between January 1987 and December 2010, and reported risk equations for survival after myocardial infarction and stroke only, in 1839 adults with type 1 diabetes. Thus, their conclusion that prior SHE was not associated with an increased risk of subsequent CVD was based only on myocardial infarction and stroke. Our study used a composite outcome comprising occurrence of hospital admissions with a primary diagnosis of non-fatal AMI, coronary heart disease, non-fatal stroke, atrial fibrillation or heart failure, or recorded cardiovascular death. The different methodology and the wider selection of CVD-related primary diagnoses is likely to influence comparisons with earlier studies, as each of these primary diagnoses have been associated with increased hospitalisation for adults with type 1 diabetes using BGM, and with reductions in relative rates for isCGM users [15]. An assessment of atherosclerosis in type 1 diabetes in the DCCT cohort found that SHE is associated with increased coronary artery calcification scores for individuals with HbA_1c_ <58 mmol/mol (<7.5%) [26]. During acute hypoglycaemia, heart rate, systolic BP and blood flow (which are associated with risk of CVD) all increase [27]. The association with atherosclerotic disease is also supported by one study showing that repeated hypoglycaemia in type 1 diabetes, including in individuals with impaired awareness of hypoglycaemia, may be an aggravating factor for markers of preclinical atherosclerosis [6]. Prior SHE may also be a marker for increased frequency of non-severe hypoglycaemia, which has a cumulative effect on cardiac structures, as hypoglycaemia is implicated in cardiac arrythmias in type 1 diabetes [28–30]. More directly, studies have shown that acute hypoglycaemia can have proinflammatory sequelae that may contribute to atherogenesis in type 1 diabetes [16, 17]. Repeated hypoglycaemia is also associated with enhanced coagulation, oxidative stress, vascular inflammation, endothelial dysfunction and platelet activation [31], which are features of CVD. However, we should point out that isCGM has also been shown to reduce time in hyperglycaemia and glucose variability [12, 13], which are not recorded in the NDR. The influence of these variables on CVD complications should be examined in future trials.

The association of prior SHE with increased CVD outcomes in type 1 diabetes is a clinically important finding of the current and previous studies [4, 15] using the NDR and NPR datasets. Notably, our findings do not prove a causal link between severe or recurrent hypoglycaemia and CVD complications but only an association between the two phenomena. However, in our study, the use of isCGM was certainly associated with a lower risk of CVD complications as a composite outcome in adults with type 1 diabetes, both for those with and without a prior SHE, compared with use of BGM, with the reduction being most noticeable in those with a prior SHE (Fig. 2).

### Strengths and limitations

This retrospective cohort study has limitations. Initiation of isCGM is a key variable in our analysis, but other factors may have influenced the outcomes. For example, it is possible that new isCGM users received device-related education at the point of initiation, which may have improved diabetes or cardiovascular self-care behaviours that we cannot control for. Equally, the NDR does not contain information on glycaemic variability or time in hyperglycaemia, so their influence cannot be assessed here. When comparing baseline characteristics of isCGM users against BGM control participants before weighting (ESM Table 2), there was an age differential between isCGM users and BGM control participants (mean 50.08 vs 66.21 years) and a minority of adults with type 1 diabetes were CSII users (12.6% of isCGM users and 2.3% of BGM control participants). Both age and CSII therapy may be associated with reduced CVD mortality compared with multiple daily injections with insulin [32], and may represent a confounding factor within the composite CVD complications reported. Likewise, the baseline prevalence of ischaemic heart disease was lower among isCGM users (8.6%) than BGM control participants (15.7%). However, after PS-IPTW, the weighted standard differences between the groups with regard to age (−0.172), use of CSII (0.167) and ischaemic heart disease (−0.135) were all below 0.20 (ESM Table 2), which suggests adequate balancing of the two groups in each case, with only a small degree of residual confounding after weighting that is unlikely to affect the outcomes in a meaningful way [22]. The same may be argued for the baseline prevalence of kidney disease, where the weighted standardised difference for serum creatinine between the cohorts was above 0.20. However, a double robust method, using additional variable adjustment in the regression analysis, was used to minimise the effect of this remaining imbalance, and other measures of kidney function were adequately balanced. Thus, we believe that kidney disease is unlikely to influence the outcomes meaningfully. The majority of other covariates between isCGM and BGM users were associated with weighted standard differences below 0.10, with negligible confounding influence (ESM Table 2). PS-IPTW also indicates that the isCGM and BGM cohorts were well matched for baseline covariates whether they had a prior SHE or not (Table 1, ESM Tables 3 and 4). These analytical validations increase the internal confidence of this comparative study, although we acknowledge that this is a limitation for the generalisability of our outcomes. Although the PS-IPTW assessment gives us confidence that the BGM and isCGM cohorts are adequately balanced, the NDR provides no information as to why a person with type 1 diabetes does not use either isCGM or real-time CGM, which is a limitation for interpretation of the comparative outcomes. As we have used PS-IPTW to balance baseline characteristics for BP and lipid profiles, along with physical activity measures and any overt ischaemic heart disease, no information on CVD co-medications is included. As the NDR does not report specific dates related to SHEs, we cannot delineate the exact time periods between occurrence of SHEs and CVD complications, which is a limitation. Baseline characteristics related to ethnicity, education or indices of social deprivation are not available in the NDR or NPR, so the influence of these factors on admission rates for CVD complications cannot be assessed. These limitations allow us to make clear associations between the use of isCGM with regard to CVD complications in adults with type 1 diabetes but do not allow us to draw definitive conclusions regarding causality. Strengths of our study include the large unselected population, with almost complete coverage of the adult type 1 diabetes population in Sweden, together with the availability of a matched control group of type 1 diabetes patients with previous SHE who used BGM rather than isCGM or real-time CGM systems. This has allowed us to minimise the risk of residual confounding and to mitigate the impact of other factors on glycaemic control over the study period that are independent of isCGM use.

### Conclusions

This retrospective cohort study shows that a prior SHE in adults with type 1 diabetes in Sweden is associated with an approximate doubling of risk of hospitalisation for CVD complications, compared to adults with type 1 diabetes and no prior SHE. Use of isCGM by adults with type 1 diabetes is associated with a 78% relative reduction in the rate of hospitalisation for CVD complications following a SHE, compared to adults with type 1 diabetes who use BGM. These outcomes illustrate the impact of SHE on the incidence of CVD episodes requiring hospitalisation, which may impact the long-term cost-effectiveness of using isCGM for the management of glucose levels in type 1 diabetes.

## Supplementary Information

Below is the link to the electronic supplementary material.

ESM (PDF 405 KB)

## Data Availability

Data not presented in the analysis are available on request from the authors

## Abbreviations

AMI: Acute myocardial infarction
BGM: Blood glucose monitoring
CGM: Continuous glucose monitoring
CSII: Continuous subcutaneous insulin infusion
isCGM: Intermittently scanned continuous glucose monitoring
NDR: National Diabetes Register
NPR: National Patient Register
PS-IPTW: Propensity score-based inverse probability of treatment weighting
SHE: Severe hypoglycaemia event

## Authors’ relationships and activities

KE-O has received fees for lecturing and/or honoraria for consulting from Sanofi, Novo Nordisk, Eli Lilly and Abbott Diabetes Care. DN has received honoraria from Abbott for the work in this study. TS has received compensation for serving on scientific advisory boards and honoraria for consultancy from Biogen, and speaker honoraria from Novartis, and is employed by Synergus RWE, who received reimbursement from Abbott to perform the analysis. MK is also employed by Synergus RWE, who received reimbursement from Abbott to perform the analysis. FL-G is an employee of Abbott Laboratories Ltd. AS is an employee of Abbott Diabetes Care. JB has received honoraria for consulting and/or lecture fees from Abbott Diabetes Care, Nanexa, Nordic InfuCare and Novo Nordisk.
