# Supplementary material for: Severe hypoglycaemia is associated with increased risk of adverse cardiovascular complications in adults with type 1 diabetes: risk mitigation using intermittently scanned continuous glucose monitoring
Source: Diabetologia. 2025 Apr 24;68(8):1647–56. doi: 10.1007/s00125-025-06438-y (PMC12245946; doi:10.1007/s00125-025-06438-y)
Supplement: Supplementary file 1 — ESM (PDF 405 KB) [file 125_2025_6438_MOESM1_ESM.pdf]

Electronic Supplementary Material

Severe hypoglycaemia is associated with increased risk of adverse cardiovascular complications in adults with type 1 diabetes: risk mitigation using intermittently scanned continuous glucose monitoring

Katarina Eeg-Olofsson[1], David Nathanson[2], Tim Spelman[3], Mattias Kyhlstedt[3], Alexander Seibold[4], Fleur Levrat-Guillen[5], Jan Bolinder[2].

1. Sahlgrenska University Hospital and Department of Molecular & Clinical Medicine, University of Gothenburg, Sweden.
2. Department of Medicine, Karolinska University Hospital Huddinge, Karolinska Institute, Stockholm, Sweden
3. Synergus RWE AB, Stationsvägen 18, 184 50 Åkersberga, Sweden
4. Abbott Diabetes Care, Wiesbaden, Germany
5. Abbott Laboratories Ltd, Maidenhead, UK

ESM Table 1. Coding of cardiovascular complications of diabetes for registry-based identification using ICD 10 codes and hospital procedure codes (KVÅ).

Diabetes complications and concomitant conditions	ICD 10 codes or procedural codes
**Primary diagnosis included in CVD outcomes**	
Acute myocardial infarction	I21
Ischaemic heart disease	I22-I25
Cardiovascular disease	I21, I61, I62, I163, I164
Atrial fibrillation	I48
Heart failure	I50
Stroke	I61, I63, I64, I67.9
Fatal CHD	I20, I21, I22, I23, I25, I24
Fatal CVD	I20, I21, I22, I23, I25, I24, I61, I63, I64

Note: KVÅ - Klassifikation av vårdåtgärder [Classification of health care interventions; NBHW]

ESM Table 2. Baseline characteristics of adult incident isCGM users and BGM controls with type 1 diabetes who have a prior SHE

Characteristic at baseline registration in dataset	Category	isCGM users (n=970)	BGM controls (n=343)	Standardised difference	Weighted baseline characteristics* isCGM users	Weighted baseline characteristics* BGM controls	Weighted standardised difference
Age (years) - mean (SD)		50.08 (18.03)	66.21 (14.90)	-0.975	56.70 (16.54)	59.53 (15.01)	-0.172
Sex - n (%)	Female	405 (41.8)	149 (43.4)	-0.034	411 (42.4)	142 (41.4)	-0.013
	Male	565 (58.3)	194 (56.6)		559 (57.6)	201 (58.6)	
BMI - mean (SD)	kg/m^2	26.19 (3.32)	26.98 (3.70)	-0.224	26.41 (3.45)	26.32 (3.65)	0.025
HbA_{1c} - mean (SD)	mmol/mol	63.1 (13.9)	63.5 (13.1)	0.007	63.4 (12.9)	63.2 (13.4)	0.017
	%	7.92 (1.28)	7.96 (1.20)		7.95 (1.18)	7.93 (1.23)	
Diabetes duration (years) - mean (SD)	Years	24.84 (16.53)	28.44 (18.83)	-0.203	25.59 (16.94)	27.29 (17.52)	-0.100
Insulin pump users – n (%)		122 (12.6)	8 (2.3)	0.397	94 (9.7)	23 (6.7)	0.167
SBP - mean (SD)	mmHg	128.28 (12.58)	134.02 (15.72)	-0.403	130.98 (13.44)	133.22 (13.57)	-0.142
DBP - mean (SD)	mmHg	74.59 (7.54)	72.18 (9.15)	0.287	74.64 (8.01)	73.55 (8.05)	0.136
LDL - mean (SD)	mmol/L	2.47 (0.66)	2.39 (0.58)	0.137	2.47 (0.65)	2.38 (0.69)	0.135
HDL - mean (SD)	mmol/L	1.59 (0.39)	1.56 (0.35)	0.092	1.59 (0.51)	1.57 (0.52)	0.038
Triglycerides - mean (SD)	mmol/L	1.16 (0.56)	1.34 (0.54)	-0.321	1.23 (0.59)	1.31 (0.61)	-0.132
Total cholesterol - mean (SD)	mmol/L	4.47 (0.75)	4.43 (0.68)	0.066	4.46 (0.81)	4.42 (0.79)	0.050
Creatinine - mean (SD)	µmol/L	79.88 (31.12)	92.18 (48.04)	-0.304	82.01 (32.10)	89.23 (31.82)	-0.229
eGFR - mean (SD)	$mL/min/1.73\ m^2$	90.46 (24.09)	76.44 (21.15)	0.618	87.86 (25.62)	84.38 (24.30)	0.139
Albuminuria - *n* (%)	No	869 (89.6)	288 (84.0)	-0.175	864 (89.1)	292 (85.1)	-0.109
	Previous	19 (2.0)	8 (2.3)		20 (2.1)	7 (2.0)	
	Microalbuminuria	60 (6.2)	32 (9.3)		62 (6.4)	28 (8.2)	
	Macroalbuminuria	22 (2.3)	15 (4.4)		24 (2.5)	16 (4.7)	
Physical activity - *n* (%)	Never	106 (10.9)	40 (11.7)	0.157	107 (11.0)	38 (11.1)	0.115
	Less than once a week	124 (12.8)	49 (14.3)		126 (13.0)	45 (13.1)	
	Regularly - 1-2 times a week	217 (22.4)	63 (18.4)		214 (22.1)	66 (19.2)	
	Regularly - 3-5 times a week	275 (28.4)	75 (21.9)		269 (27.7)	81 (23.6)	
	Daily	248 (25.6)	116 (33.8)		254 (26.2)	113 (32.9)	
Ischaemic heart disease - *n* (%)		83 (8.6)	54 (15.7)	0.221	96 (9.9)	44 (12.8)	-0.135
Retinopathy - *n* (%)		685 (70.6)	226 (65.9)	-0.102	674 (69.5)	233 (67.9)	-0.041
Stroke - *n* (%)		64 (6.6)	27 (7.9)	0.049	68 (7.0)	25 (7.3)	0.002
Smoker - *n* (%)		134 (13.8)	28 (8.2)	-0.181	123 (12.7)	34 (9.9)	-0.116

* Cohort numbers after weighting not shown. isCGM, intermittently-scanned continuous glucose monitoring; BGM, blood-glucose monitoring; SHE, severe hypoglycaemia event; BMI, body-mass index; DBP, diastolic blood pressure; SBP, systolic blood pressure; LDL, low-density lipoprotein; HDL, high-density lipoprotein

ESM Table 3. Baseline characteristics for all adults with type 1 diabetes, with and without a prior SHE episode (isCGM users only)

Characteristic at baseline registration in dataset	Category	isCGM users with prior SHE (n=970)	IsCGM users without prior SHE (n=10,852)	Standardised difference	Weighted baseline characteristics		Weighted standardised difference
					isCGM users with prior SHE (n=970)	isCGM users without prior SHE (n=10,852)	
Age (years) - mean (SD)		50.08 (18.03)	50.31 (18.14)	-0.013	50.11 (18.04)	50.25 (18.11)	-0.008
Sex - *n* (%)	Female	405 (41.8)	4497 (41.4)	0.006	404 (41.6)	4502 (41.5)	0.004
	Male	565 (58.3)	6355 (58.6)		566 (58.4)	6350 (58.8)	
BMI - mean (SD)	kg/m^2	26.19 (3.32)	26.21 (3.72)	-0.026	26.19 (3.41)	26.20 (3.68)	-0.003
HbA_{1c} - mean (SD)	mmol/mol	63.1 (13.9)	62.7 (13.9)	0.005	63.1 (14.0))	62.8 (13.9)	0.016
	%	7.92 (1.28)	7.89 (1.28)		7.92 (1.29)	7.90 (1.28)	
Diabetes duration (years) - mean (SD)	Years	24.83 (16.53)	22.03 (16.50)	0.170	24.31 (16.51)	23.06 (16.51)	0.076
Insulin pump users – *n* (%)		122 (12.6)	1186 (10.9)	0.051			
SBP - mean (SD)	mmHg	128.28 (12.58)	128.23 (12.92)	0.003	128.27 (112.55)	128.24 (112.55)	0.000
DBP - mean (SD)	mmHg	74.59 (7.54)	74.52 (7.80)	0.009	74.58 (7.60)	74.54 (7.83)	0.005
LDL - mean (SD)	mmol/L	2.47 (0.66)	2.49 (0.68)	-0.021	2.47 (0.67)	2.48 (0.68)	-0.015
HDL - mean (SD)	mmol/L	1.59 (0.39)	1.59 (0.41)	0.001	1.59 (0.40)	1.59 (0.42)	0.000
Triglycerides - mean (SD)	mmol/L	1.16 (0.56)	1.15 (0.66)	0.011	1.16 (0.57)	1.16 (0.67)	0.000
Total cholesterol - mean (SD)	mmol/L	4.47 (0.75)	4.48 (0.78)	-0.010	4.47 (0.76)	4.47 (0.79)	0.000
Creatinine - mean (SD)	µmol/L	79.88 (31.12)	77.96 (36.03)	0.057	79.85 (31.24)	76.68 (35.81)	0.035
eGFR - mean (SD)	$mL/min/1.73\ m^2$	90.46 (24.09)	92.01 (22.89)	-0.066	90.95 (24.13)	91.46 (23.04)	-0.022
Albuminuria - *n* (%)	No	869 (89.6)	9914 (91.4)	0.055	871 (89.8)	9908 (91.3)	0.039
	Previous	19 (2.0)	144 (1.3)		17 (1.8)	148 (1.4)	
	Microalbuminuria	60 (6.2)	607 (5.6)		58 (6.0)	613 (5.6)	
	Macroalbuminuria	22 (2.3)	187 (1.7)		24 (2.5)	183 (1.7)	
Physical activity - *n* (%)	Never	106 (10.9)	897 (8.3)	-0.073	103 (10.6)	905 (8.3)	-0.058
	Less than once a week	124 (12.8)	1294 (11.9)		122 (12.6)	1304 (12.0)	
	Regularly - 1-2 times a week	217 (22.4)	2185 (20.1)		215 (22.2)	2193 (20.2)	
	Regularly - 3-5 times a week	275 (28.4)	3491 (32.2)		281 (29.0)	3485 (32.1)	
	Daily	248 (25.6)	2985 (27.5)		249 (25.7)	2965 (27.3)	
Ischaemic heart disease - *n* (%)		83 (8.6)	825 (7.6)	-0.035	81 (8.4)	831 (7.7)	-0.031
Retinopathy - *n* (%)		685 (70.6)	6849 (63.1)	-0.160	680 (70.1)	6858 (63.2)	-0.117
Stroke - *n* (%)		64 (6.6)	437 (4.0)	-0.115	62 (6.4)	450 (4.1)	-0.098
Smoker - *n* (%)		134 (13.8)	1214 (11.2)	-0.079	129 (13.3)	1219 (11.2)	-0.064

isCGM, intermittently-scanned continuous glucose monitoring; BGM, blood-glucose monitoring; SHE, severe hypoglycaemia event; BMI, body-mass index; DBP, diastolic blood pressure; SBP, systolic blood pressure; LDL, low-density lipoprotein; HDL, high-density lipoprotein

ESM Table 4. Baseline characteristics for all adults with type 1 diabetes, with and without a prior SHE episode (BGM users only)

Characteristic at baseline registration in dataset	Category	BGM users with prior SHE (n=343)	BGM users without prior SHE (n=2664)	Standardised difference	Weighted baseline characteristics		Weighted standardised difference
					BGM users with prior SHE (n=343)	BGM users without prior SHE (n=2664)	
Age (years) - mean (SD)		66.21 (14.90)	62.89 (16.52)	0.211	65.78 (15.04)	63.91 (16.11)	0.120
Sex - *n* (%)	Female	149 (43.4)	1123 (42.2)	0.026	147 (42.9)	1129 (42.4)	0.018
	Male	194 (56.6)	1541 (57.9)		196 (57.1)	1535 (57.6)	
BMI - mean (SD)	kg/m^2	26.98 (3.70)	26.94 (4.10)	0.012	26.97 (3.81)	26.95 (3.97)	0.005
HbA_{1c} – mean (SD)	mmol/mol	63.5 (13.1)	61.5 (13.5)	0.146	63.0 (13.1)	62.1 (13.1)	
	%	7.96 (1.20)	7.78 (1.23)		7.91 (1.20)	7.83 (1.20)	0.067
Diabetes duration (years) - mean (SD)	Years	28.44 (18.83)	23.06 (17.36)	0.297	27.15 (18.44)	24.94 (17.95)	0.121
Insulin pump users – *n* (%)		8 (2.3)	52 (2.0)	0.026	8 (2.3)	55 (2.1)	0.019
SBP - mean (SD)	mmHg	134.02 (15.72)	132.66 (13.66)	0.092	133.86 (15.54)	133.02 (14.36)	0.056
DBP - mean (SD)	mmHg	72.18 (9.15)	74.12 (8.56)	-0.219	72.81 (8.88)	73.89 (8.79)	-0.122
LDL - mean (SD)	mmol/L	2.39 (0.58)	2.47 (0.67)	-0.128	2.41 (0.60)	2.44 (0.67)	-0.047
HDL - mean (SD)	mmol/L	1.56 (0.35)	1.54 (0.38)	0.055	1.56 (0.36)	1.55 (0.39)	0.027
Triglycerides - mean (SD)	mmol/L	1.34 (0.54)	1.39 (0.73)	-0.078	1.35 (0.55)	1.38 (0.70)	-0.048
Total cholesterol - mean (SD)	mmol/L	4.43 (0.68)	4.48 (0.83)	-0.077	4.44 (0.69)	4.47 (0.77)	-0.041
Creatinine - mean (SD)	µmol/L	92.18 (48.04)	86.52 (42.54)	0.125	91.45 (47.71)	88.69 (43.96)	0.060
eGFR - mean (SD)	$mL/min/1.73\ m^2$	76.44 (21.15)	80.96 (22.32)	-0.208	77.63 (21.26)	80.24 (22.41)	-0.119
Albuminuria - *n* (%)	No	288 (84.0)	2250 (84.5)	0.028	288 (84.0)	2248 (84.4)	0.022
	Previous	8 (2.3)	55 (2.1)		7 (2.0)	56 (2.1)	
	Microalbuminuria	32 (9.3)	282 (10.6)		34 (9.9)	278 (10.4)	
	Macroalbuminuria	15 (4.4)	77 (2.9)		14 (4.1)	82 (3.1)	
Physical activity - *n* (%)	Never	40 (11.7)	360 (13.5)	-0.028	42 (12.2)	356 (13.4)	-0.024
	Less than once a week	49 (14.3)	352 (13.2)		48 (14.0)	355 (13.3)	
	Regularly - 1-2 times a week	63 (18.4)	463 (17.4)		61 (17.8)	468 (17.6)	
	Regularly - 3-5 times a week	75 (21.9)	640 (24.0)		77 (22.4)	636 (23.9)	
	Daily	116 (33.8)	849 (31.9)		115 (33.5)	849 (31.9)	
Ischaemic heart disease - *n* (%)		54 (15.7)	393 (14.8)	-0.028	52 (15.2)	396 (14.9)	-0.024
Retinopathy - *n* (%)		226 (65.9)	1586 (59.5)	-0.132	223 (65.0)	1592 (59.8)	-0.114
Stroke - *n* (%)		27 (7.9)	181 (6.8)	-0.041	26 (7.6)	184 (6.9)	-0.036
Smoker - *n* (%)		28 (8.2)	334 (12.5)	0.144	31 (9.0)	322 (12.1)	0.119

isCGM, intermittently-scanned continuous glucose monitoring; BGM, blood-glucose monitoring; SHE, severe hypoglycaemia event; BMI, body-mass index; DBP, diastolic blood pressure; SBP, systolic blood pressure; LDL, low-density lipoprotein; HDL, high-density lipoprotein

ESM Figure 1. The identification and selection of new incident users of isCGM with type 1 diabetes and matched controls with type 1 diabetes using BGM

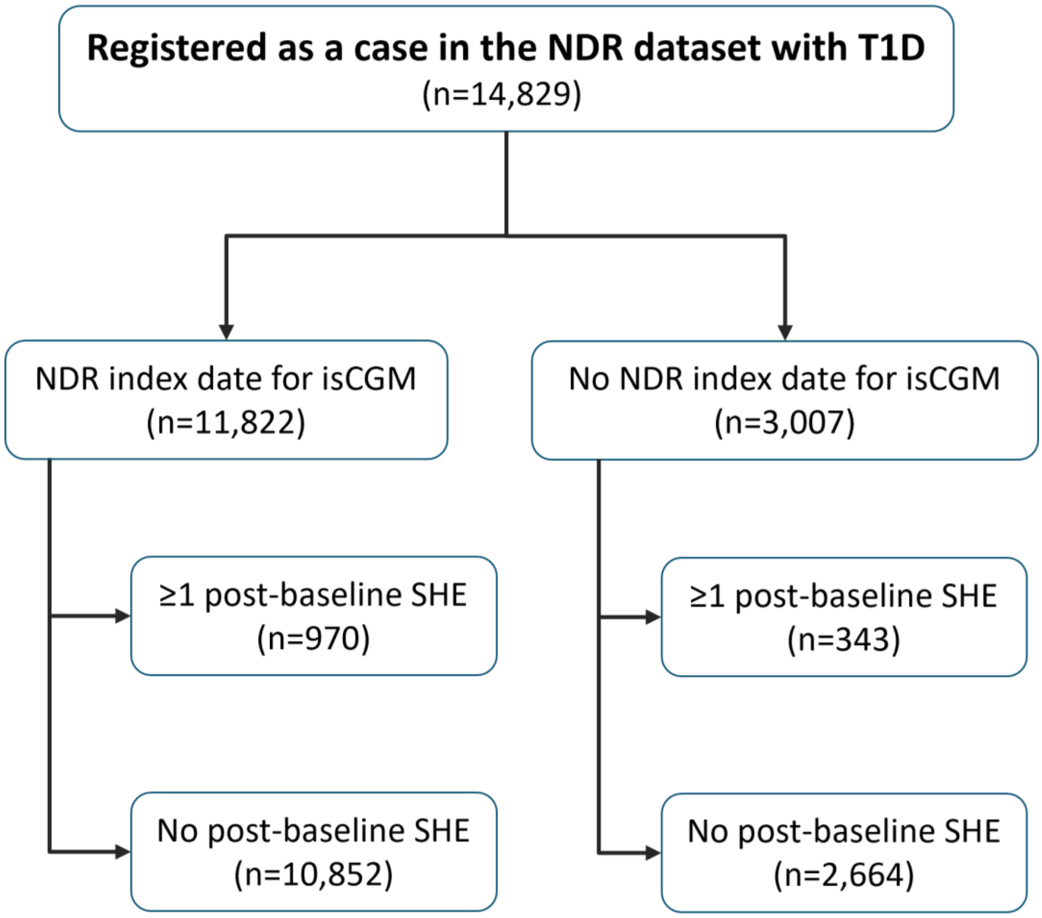

isCGM, intermittently-scanned continuous glucose monitoring; NDR, national Diabetes Register; SHE. Severe hypoglycaemia event; T1D, type 1 diabetes

ESM Figure 2. Hospitalisation rate for AMI, stroke or CV death in adults with T1D with or without prior SHE, by glucose monitoring method

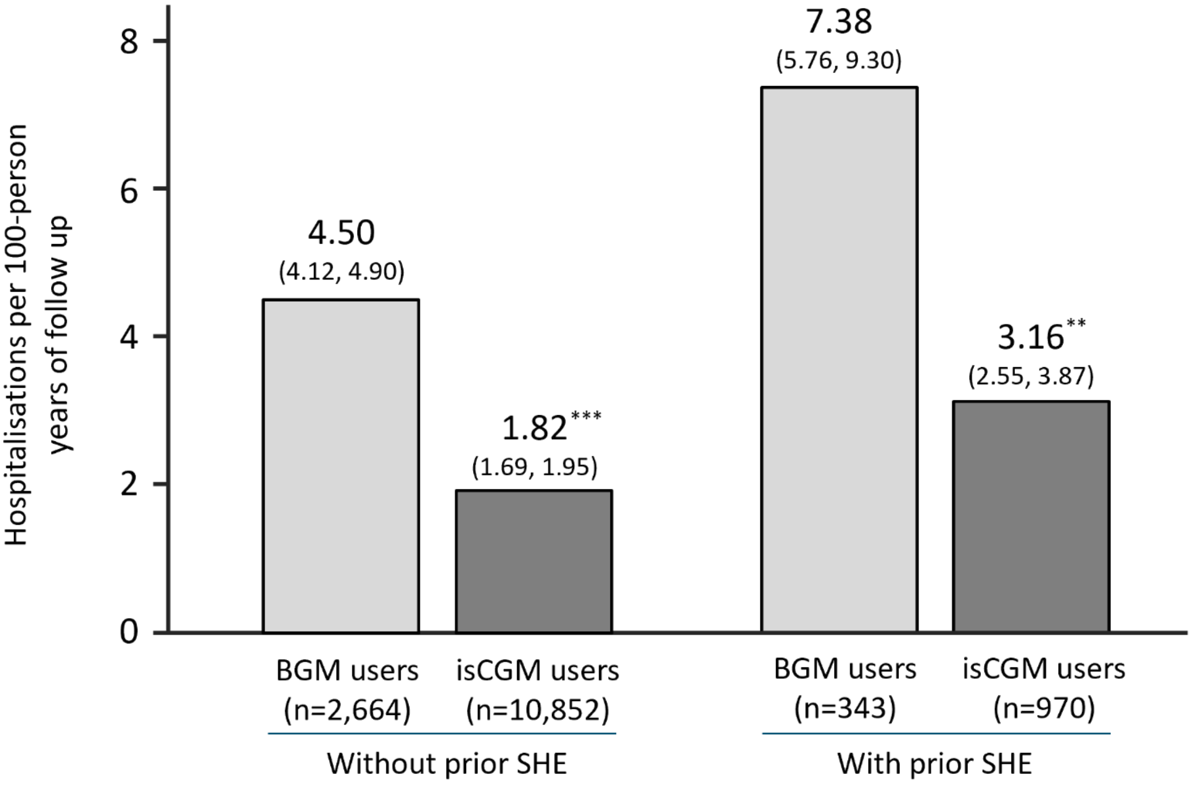

Negative binomial regression was used to compare the rates of a composite outcome of AMI, stroke or CV death following SHE in isCGM users relative to BGM users. Propensity-score based Inverse Probability of Treatment Weighting (PS-IPTW) was used to adjust for differences in confounders at baseline between the groups. Figure shows relative rate calculations with 95% confidence intervals. **$p<0.01$, ***$p<0.001$

Abbreviations. AMI, acute myocardial infarction, BGM, blood glucose monitoring; CV, cardiovascular; isCGM, intermittently scanned continuous glucose monitoring; SHE, severe hypoglycaemia event; T1D, type 1 diabetes
